# Apple Configurator 2 (version 2.3)

**DOI:** 10.5195/jmla.2018.325

**Published:** 2018-01-02

**Authors:** Lara Lasner-Frater

## OVERVIEW

Apple Configurator 2 (AC2) [1] is a free mass-deployment utility that allows you to update multiple iPads, iPhones, iPod Touch devices, and Apple TVs at the same time, including apps, website links, iBooks, and software updates. Such a tool can be useful for libraries that lend iPads to their users. This review is based on the author’s experience in a library that circulated fifteen iPads to graduate students in the medical field. About ninety-five free apps (in addition to preloaded Apple apps), thirty website icons, and various folders were installed using AC2 from a Master iPad backup.

The iPads were “unsupervised,” which meant students could download their own apps and change settings if needed. When the iPads were returned, they were erased and reimaged from the backup on the Master iPad. No additional mobile device management (MDM) tools were used. Apps went directly from Apple’s volume purchase program (VPP) to AC2. Unfortunately, as further discussed in this review, AC2 is plagued with issues: it is difficult and confusing to set up, requires multiple steps, and is prone to errors.

AC2 version 2.3 was set up on a circa 2012 MacBook Pro using the El Capitan Mac operating system (Mac OS). The laptop is dedicated solely to AC2 and was set up by the author, who has basic skills with Mac computers but no prior experience with Apple Configurator. Note that while this review is based on AC2 version 2.3, Apple recently released version 2.4, which requires the Sierra version of Mac OS.

The AC2 manual is linked from the iTunes store. It is not user friendly, as it does not allow you to download the entire manual, nor can you browse the entire manual at once [2]. Setup required using trial and error, searching the web, watching third-party tutorials, and contacting Apple and other librarians.

## FEATURES

AC2 allows you to add and manage apps, web icons, documents, and iBooks content (electronic books and portable document format files [PDFs]). You must first set up a Master iPad or a Blueprint with the desired content; then, you can deploy that content to up to thirty devices. You can create “supervised” iPads, a feature that prevents patrons from changing settings, as well as separate profiles for different user groups (e.g., faculty, students). AC2 allows you to add background images and modify the iPad home screen, for example, to put a heavily used app on the first page or put similar apps in the same folder. You can use AC2 alone or with MDM applications.

According to the AC2 entry in the iTunes store [1], version 2.4 includes changes to some features that may be of interest to librarians. For example, it allows you to install PDFs, ePubs, and iBooks; restart or shutdown iOS devices; and restrict joining WiFi networks.

## INSTALLATION AND SETUP

As with most Apple apps, AC2 has to be downloaded from the iTunes store. It is not available for PCs, nor will it work with other non-Apple products. An Apple VPP account is required to add apps using AC2—even if you are using only free apps [3]—though this requirement is not mentioned in the AC2 documentation. Apple can often make getting VPP accounts difficult, especially for institutions with multiple libraries, as Apple tends to give out only one account per school. When applying for a VPP account, Apple requires a Dun and Bradstreet number [4]. Once you have an account set up, you can go to the VPP website and purchase apps for your iPads; even free apps need to be “purchased.” Sign on to both VPP and AC2, so that apps ordered on VPP will automatically be added to the AC2 app folder. Note that some iOS apps do not work with AC2.

The AC2 interface is easy to use. It is easy to change the device name, add or remove apps, and erase or load a blueprint or a backup. In addition, AC2 indicates if your OS and apps are out of date when you plug in an iPad, and it automatically updates apps from VPP to the newest version.

## BLUEPRINT VERSUS MASTER IPAD

You can set up your iPads in two ways: You can create a Blueprint or a Master iPad. A Blueprint requires you to set up everything in AC2. A Master iPad requires you to set up everything on an iPad, then back it up on AC2. You can then apply that backup to other iPads. Both options have advantages and disadvantages, as shown in Table 1.

**Table 1:** Advantages and disadvantages of using a Blueprint versus Master iPad

	Advantages	Disadvantages
Blueprint	Set up directly on AC2. No back up iPad needed.	Is difficult to set up.
	Need to create multiple blueprints to do different tasks (e.g., one blueprint to restore and update the IOS and another to deploy).	Is extremely difficult to set up web shortcuts/icons. Requires creating a profile and icon, then copying and pasting the link.
	Install everything at once onto iPads including setup and apps.	
Master iPad	Set up an iPad exactly how you want it.	Keeps one iPad out of circulation.
	Creates multiple backups.	Requires manual setup of an iPad.
	Can set up web icons easily. Save the web page on your Master iPad, and when you back up, it will save the icon and the location.	Will install setup, folders, and web icons, but apps have to be installed manually.

## PROBLEMS

While the interface is nicely designed, deploying iPads using AC2 was extremely difficult. Through trial and error, it eventually evolved into a long thirteen-step process. When setting up AC2, I highly recommend documenting each step. Not only is the process long, but errors are frequent. Sometimes the system freezes or takes a long time to process errors.

These errors often required lengthy research into the cause. Reimaging using a backup of a Master iPad required loading all the apps again, which on an old MacBook sometimes took up to two hours per device—if there were no errors. Reimaging also required multiple steps and frequently generated errors. The process could not be left unattended, because it required periodic checks for errors.

## ALTERNATIVES

Many companies offer MDM products (e.g., GroundControl, MacProfessional, Meraki, and Airwatch). Those products do not all have the same features that AC2 has, and they are not free.

## SUMMARY

AC2 is fine for small iPad collections, but using it to manage larger ones requires considerable time, knowledge, and effort. Yet this product was designed to save time. The product is difficult to set up, and using advanced features requires extensive knowledge of how the program works. Better documentation and video tutorials would be helpful. As of this writing, the author has still not updated to AC2 version 2.4 because of the time and effort required to set up 2.3.
